# Molecular survey of mammalian orthoreovirus and Rotavirus A in Italian dogs and cats

**DOI:** 10.3389/fvets.2026.1798401

**Published:** 2026-04-29

**Authors:** Mery Campalto, Elisa Mazzotta, Francesca Crescenti, Marilena Carrino, Alice Fincato, Patrizia Danesi, Lara Cavicchio, Laura Gagliazzo, Letizia Ceglie, Alda Natale

**Affiliations:** Istituto Zooprofilattico Sperimentale delle Venezie, Legnaro, PD, Italy

**Keywords:** cat, dog, mammalian orthoreovirus, rotavirus, zoonosis

## Abstract

Mammalian orthoreoviruses (MRVs) and Rotavirus A (RVA) are double-stranded RNA viruses with zoonotic potential that can infect a wide range of mammalian hosts, including companion animals. Data on their circulation in dogs and cats remain limited worldwide. Building on a previous study conducted on shelter-housed dogs and cats (RC IZSVE 12/19), this study investigated the presence of MRV and RVA in owned dogs and cats primarily from Northeastern Italy. Between 2023 and 2025, a total of 648 fecal samples from 278 dogs to 370 cats were collected within a research project (RF MRV 21) and analyzed by multiplex rRT-PCR targeting conserved regions of the MRV L1 and RVA NSP3 genes. MRV-positive samples were subjected to viral isolation in Vero cells. Samples were also examined for Canine and Feline Coronavirus (CCoV and FCoV), Canine parvovirus type 2 and Feline panleukopenia virus (CPV-2 and FPV) and intestinal parasites using standard copromicroscopic methods as part of differential diagnosis for gastroenteric disorders. MRV RNA was detected in 2 dogs (0.7%) and 3 cats (0.8%). RVA was identified in 4 dogs (1.4%) and 7 cats (1.9%). MRV isolation was attempted from all positive samples (dogs: 2/278, 0.72%; cats: 3/370, 0.81%), as well as from MRV-positive samples identified in the previous study (dogs: 1/257, 0.39%; cats: 11/388, 2.83%), and was successfully achieved for seven feline (7/14, 50.00%) and one canine samples (1/3, 33.33%). For animals positive for MRV and/or RVA, if symptomatic, the more common differential diagnoses were considered. This study provides comprehensive molecular evidence of MRV and RVA circulation in dogs and cats in Italy. The detection and successful isolation of MRVs from both species highlight their potential role in viral ecology and the importance of continued surveillance to assess zoonotic risks at the human-animal interface.

## 1 Introduction

Mammalian orthoreoviruses (MRVs) are non-enveloped, double-stranded RNA viruses belonging to the genus *Orthoreovirus* within the family *Spinareoviridae*. MRVs were first identified in humans in the 1950s (1) and are typically associated with mild or asymptomatic gastroenteric (2–7) and respiratory diseases (8–10) in both humans and animals. However, MRVs have also been implicated in more severe conditions, including

encephalopathy (11) and meningitis (12, 13). Their genome, approximately 23.5 kilobases (kb) in length, is divided into ten segments, grouped by electrophoretic mobility into three size classes: large (L1–L3), medium (M1–M3), and small (S1–S4) (1). These segments encode eight structural proteins (λ1, λ2, λ3, μ1, μ2, σ1, σ2, and σ3) and four non-structural proteins (μNS, μNSC, σNS, and σ1s). MRVs are classified into four serotypes, MRV1 to MRV4, based on neutralization and hemagglutination inhibition assays targeting σ1 (14, 15). Notably, the S1 gene encodes both the structural σ1 protein, crucial for cell attachment, receptor binding, and virulence, and the non-structural σ1s protein (1). The segmented genome facilitates genetic reassortment and intragenic rearrangements, driving viral evolution and the emergence of novel strains with unpredictable biological characteristics, raising significant concerns about their zoonotic potential. This genetic plasticity is reflected in the absence of strict species barriers, as MRVs have been reported in humans (3, 4, 16, 17), livestock (18–22), wildlife (23–25) and pets (7, 26–30). Despite the growing importance of MRVs as emerging pathogens, little is known about their circulation in companion animals such as dogs and cats. This knowledge gap is particularly relevant considering the increasingly close contact between humans and pets, which may facilitate zoonotic transmission. To date, no complete genome sequences from either dogs or cats have been reported at the national or international level, and in Italy, MRVs have only been partially characterized in a single canine case associated with fatal diarrhea (7). This gap underscores the need for comprehensive epidemiological studies to assess the presence and diversity of MRVs in companion animals. Rotaviruses (RVs), members of the genus *Rotavirus* in the family *Sedoreoviridae*, are also non-enveloped, double-stranded RNA viruses. RVs, first discovered in animals in the 1960s (31) and classified within the same order (*Reovirales*) as MRVs (32), share a similar evolutionary propensity. Their genome consists of 11 segments (~18.5 kb) encoding six structural proteins (VP1–VP4, VP6, VP7) and five or six non-structural proteins (NSP1–NSP5/6). Among the eleven recognized RV serogroups (32), Rotavirus A (RVA) is the most prevalent and clinically significant, being a major cause of acute gastroenteritis in both humans and a wide range of animal hosts worldwide (33). The segmented nature of the RVA genome similarly promotes genetic reassortment events that can give rise to novel viral strains capable of crossing species barriers (34, 35). Despite the availability of vaccines in human, RVA outbreaks continue to occur, frequently driven by the emergence of novel or reassortant strains. Increasing evidence supports the occurrence of interspecies transmission of RVA between companion animals and humans, with potential implications for public health due to zoonotic and reassortment events (34–38).

Several studies suggest that MRV and RVA may contribute to multifactorial disease processes, particularly in young animals, where co-infections with other enteric pathogens can exacerbate clinical severity and enhance viral shedding. Although the pathogenic role of MRV and RVA remains incompletely defined, MRV has been detected in dogs in association with pneumonia, upper respiratory tract disease, and enteritis, whereas RVA is more frequently identified in diarrheic puppies, often in conjunction with Canine parvovirus type 2 (CPV-2) or Canine coronavirus (CCoV) (26, 30, 39–41). In cats, both viruses have been isolated from animals presenting with gastrointestinal, respiratory, or, occasionally, neurological signs, as well as from clinically healthy individuals, suggesting that their pathogenic potential may be context dependent and influenced by host-related factors and the presence of co-infecting agents (42).

Given their frequent and close interaction with humans, dogs and cats may act as reservoirs or intermediaries for zoonotic transmission of both MRV and RVA. However, the current lack of epidemiological data, particularly regarding MRVs in companion animals, limits our understanding of their potential role in viral ecology and public health risk. Preliminary results obtained in Northeastern Italy from dogs and cats housed in animal shelters (RC IZSVE 12/19) (43), a high-risk group due to overcrowding, frequent pathogen exposure, and suboptimal health conditions, revealed a low but not negligible circulation of MRV and RVA in both species. Based on these findings, we hypothesized that MRV and RVA also circulate in owned dogs and cats, which represent a large and understudied population in close contact with humans and therefore potentially relevant for zoonotic transmission. The objective of this study was to investigate the presence of MRV and RVA in owned dogs and cats in Northeastern Italy (RF MRV 21) through molecular detection.

## 2 Materials and methods

### 2.1 Sampling

From April 2023 to December 2025, an observational cross-sectional study was conducted in which fecal samples were collected from domestic dogs and cats through collaboration with veterinary clinics and independent veterinarians. The number of samples to be collected was estimated in 300 samples per species considering the unknown prevalence of the target pathogens, and all eligible samples obtained during the study period were included accordingly. Most samples originated from Northeastern Italy, particularly the Veneto region, with a limited number obtained from other regions including Lombardia, Emilia-Romagna, Marche, Toscana, and Abruzzo.

Samples lacking owner consent, with incomplete sample submission, or with insufficient material for analysis were excluded from the study.

At the time of sample collection, epidemiological and clinical metadata, including sex, neutering status, age, clinical condition (asymptomatic or presenting gastrointestinal signs), and province of origin, were recorded for all enrolled owned dogs and cats.

Fecal samples were homogenized in the laboratory within 24 h of arrival, and an aliquot was prepared for coprological investigation. The remaining samples were then stored at −80 °C until further analysis.

This study received the approval of the Ethical Committee of the Istituto Zooprofilattico Sperimentale delle Venezie (CE_IZSVE 2/2023).

### 2.2 Diagnostic panel

The diagnostic panel was designed to include selected enteric pathogens commonly associated with gastrointestinal disorders in dogs and cats that can be detected through a non-invasive approach based on fecal sampling. This approach also provided clinical and diagnostic support to the veterinarians involved in sample collection. In line with the study design, which aimed to document the clinical and epidemiological context of MRV and RVA detection rather than to assess the role of co-infections, the panel was limited to enteropathogens most routinely investigated in diagnostic practice. This panel included:

- MRV
- RVA
- Canine coronavirus (CCoV) and Feline coronavirus (FCoV)
- Canine parvovirus type 2 (CPV-2) and Feline panleukopenia virus (FPV)
- Copromicroscopic test (sedimentation-flotation technique)

### 2.3 Molecular investigations

#### 2.3.1 Nucleic acid extraction

Fecal samples were suspended in 1 ml of Phosphate-Buffered Saline (PBS) supplemented with antibiotics (10,000 IU/ml penicillin G, 10 mg/ml streptomycin, 5,000 IU/ml nystatin and 0.25 mg/ml gentamicin sulfate) (PBS-A). Samples were thoroughly mixed by vortexing and briefly centrifuged. 100 microliters (μl) of each supernatant were used for viral DNA and RNA extraction using the ID Gene^®^ Mag Universal Extraction Kit (IDvet, Grabels, France) on the KingFisher™ Flex Purification System (Life Technologies, Carlsbad, CA, USA), following the manufacturer's instructions. To assess the efficiency of nucleic acid extraction and to validate negative results, universal heterologous DNA and RNA controls (Intype IC-DNA and Intype IC-RNA, Indical Bioscience GmbH, Leipzig, Germany) were added to each sample at a 1:10 ratio relative to the total elution volume and subsequently co-amplified as described by Hoffmann et al. (44). During the extraction process, an additional exogenous internal positive control provided with the VetMAX™ FIP Dual IPC Kit (Thermo Fisher Scientific, Waltham, MA, USA) was also included. The eluted nucleic acids were subsequently stored at −80 °C until use.

#### 2.3.2 Real-time PCR assays

A customized multiplex Real-time RT-PCR (rRT-PCR) assay was applied to simultaneously detect MRV and RVA, targeting a conserved region of the L1 gene (137 bp) for MRV (45) and the NSP3 gene (78 bp) for RVA (46, 47). The primers and probe targeting the MRV L1 gene were adapted from Wang et al. (45), with minor modifications introduced to improve amplification specificity in the assay format used in this study. Specifically, two nucleotide substitutions were introduced in the probe and in the reverse primer, and one nucleotide substitution in the forward primer. The final primer and probe sequences used in this study are reported in Table 1. Prior to its application, several steps were undertaken to assess the suitability of the assay for molecular detection in our dataset, including *in silico* evaluation of primer and probe binding sites across available MRV L1 sequences deposited in GenBank and testing with previously characterized MRV-positive controls. The rRT-PCR assay employed in this study was originally developed for swine samples, including fecal samples. Although formal validation was not performed on dogs' and cats' fecal samples, preliminary tests investigating the impact of this matrix on the sensitivity of the method were performed with fecal contaminated samples and the results obtained did not raise any concern contamination. The assay includes co-amplification of Intype IC-RNA. The reaction mixture comprised 9 μl of nuclease-free water, 5 μl of TaqPath one-Step Multiplex Master Mix (4X) (Life Technologies, California, USA), 1 μl of each premix (MRV, RVA, EGFP RNA), and 4 μl of denatured RNA. The multiplex rRT-PCR cycling profile consisted of UNG incubation at 25 °C for 2 min, reverse transcription at 53 °C for 10 min, polymerase activation at 95 °C for 2 min, and 40 cycles of 95 °C for 3 s, 60 °C for 30 s. Multiplex rRT-PCR was performed using a QuantStudio™ 5 Real-time PCR System instrument (Thermo Fisher Scientific, Waltham, MA, USA). Eluted RNA was tested for CCoV and FCoV using a rRT-PCR assay performed with the VetMAX™ FIP Dual IPC Kit (Thermo Fisher Scientific, Waltham, MA, USA). Eluted DNA was analyzed by Real-time PCR (rPCR) targeting a conserved region of the VP2 gene (93 bp) of CPV-2 and FPV, using an assay developed at the IZSVe (48). rPCR reaction was carried out in a final volume of 25 μl, containing 9 μL of nuclease-free water, 5 μl of quantiFast pathogen master mix (5X) (QIAGEN, Hilden, Germany), 1.5 μl of each primer (10 μm), 0.5 μl of probe (10 μm), 2.5 μl of EGFP DNA premix used as an exogenous internal amplification control, and 5 μl of DNA. Thermal cycling conditions consisted of an initial Taq polymerase activation at 95 °C for 5 min, followed by 40 cycles of denaturation at 95°C for 15 s and annealing at 59 °C for 30 s. Both molecular assays were performed using the CFX96 Deep Well Real-time PCR System (Bio-Rad Laboratories Inc., Hercules, CA, USA). Table 1 lists the primers and probes used for molecular assays. Each PCR run included a certified reference positive control, a negative extraction control to monitor potential contamination during the extraction process, and a no-template control containing nuclease-free water.

**Table 1:** Primers and probes used for molecular assays.

Pathogen	Primers/Probe identification	Primers/Probe sequence	Target	Amplicon size (bp)	Reference
MRV	MRV L1 Probe	5'- 6-FAM - TGGCAGCGDTGGATHCGBTATTC - BHQ-1 3'	L1	137	Modified Wang et al., (45)
	MRV L1 For	5'-GCGAAYTCTTCAGCRGARGAGC-3'			
	MRV L1 Rev	5'-CGTGARAAAGCRCAGCATRRAGCC-3'			
RVA	RVA7 Probe	5'- Cy5 - ATAGTTAAAAGCTAACACTGTCAAAAACCTAAA - BBQ-650 3'	NSP3	77	Otto et al., (46) Pang et al., (47)
	RVA7-1F	5'-RCATRACCCYCTATGAGCAC-3'			
	RVA NVP3-R	5'- GGTCACATAACGCCCC-3'			
Exogenous internal positive control (Intype RNA)	EGFP-HEX	5'-HEX-AGCACCCAGTCCGCCCTGAGCA-BHQ1-3'	–	132	Hoffmann et al., (44)
	EGFP-1-F	5'-GACCACTACCAGCAGAACAC-3'			
	EGFP-2-R	5'-GAACTCCAGCAGGACCATG-3'			
CPV-2/FPV	CPV-2 Probe	5'-FAM-TGGTCCTTTAACTGCATTAAATAATGTACC- TAMRA-3'	VP2	93	Carrino et al., (48)
	CPV-2 For	5'-GATCCAATTGGAGGTAAAACAGG-3'			
	CPV-2 Rev	5'-TTCTTTATCCCAAATTTGACC-3'			
Exogenous internal positive control (Intype DNA)	EGFP-HEX	5'-HEX-AGCACCCAGTCCGCCCTGAGCA-BHQ1-3'	–	–	Hoffmann et al., (44)
	EGFP-1-F	5'-GACCACTACCAGCAGAACAC-3'	–	176	
	EGFP-10-R	5'-CTTGTACAGCTCGTCCATGC-3'	–	–	

#### 2.3.3 Amplification results

MRV and RVA amplification data were analyzed using QuantStudio™ Design & Analysis Software, whereas CCoV/FCoV and CPV-2/FPV amplification data were analyzed using Bio-Rad CFX Maestro Software. A sample was considered positive when the amplification curve showed a typical exponential profile and the cycle threshold (Ct) value was ≤ 36 for MRV and ≤ 35 for RVA, < 45 for CCoV/FCoV, and ≤ 35 for CPV-2/FPV. Samples showing no amplification of viral targets or Ct values higher than the established thresholds, together with correct internal control amplification, were considered negative. Samples showing failed or abnormal internal control amplification were re-tested by repeating the PCR using undiluted and 1:10 diluted RNA. If inhibition persisted, nucleic acid extraction was repeated.

### 2.4 Viral isolation

Samples that resulted positive for MRV at the molecular assay performed within the research projects (RC IZSVE 12/19 and RF MRV 21) were subjected to virological examination. Each sample was diluted in PBS-A at a ratio of 1:3 for OP swabs and 1:10 for R swabs and fecal samples, starting from the same sample used for molecular analyses. Prior to inoculation, the diluted samples were incubated overnight at 4 °C and subsequently filtered through a 0.45 μm pore-size membrane filter. Viral isolation of MRV was attempted on African green monkey kidney (Vero) cells (ATCC CCL-81). Vero cells were maintained in Minimum Essential Medium (MEM) supplemented with 10% fetal bovine serum (FBS), 2 mm L-glutamine, 1% penicillin–streptomycin (10,000 U/ml), 0.5% gentamicin (50 mg/ml), 1% nystatin, and 0.1% amphotericin B. Subconfluent Vero cell monolayers (24 h post-passage) grown in 12.5 cm^2^ flasks were washed twice with PBS and inoculated with 400 μl of the sample mixed with 100 μl of MEM supplemented with 5 μg/ml TPCK-treated trypsin. After incubation for 1.5 h at 37 °C in a 5% CO_2_ atmosphere, 4.5 ml of post-inoculation medium supplemented with 1 μg/ml TPCK-treated trypsin was added to each flask. The flasks were incubated at 37 °C in a 5% CO_2_ atmosphere and monitored daily for cytopathic effect (CPE).

### 2.5 Copromicroscopic methods

Fecal samples were tested to identify helminth eggs as part of the differential diagnosis. The samples were processed using a standard sedimentation-flotation technique employing sodium nitrate (specific gravity 1.3). Microscopic examination of the resulting fecal floats allowed for the morphological evaluation and identification of parasite eggs based on established intestinal parasite keys (49).

### 2.6 Statistics

Sample size estimation was performed assuming an expected prevalence of 50% (used to maximize sample size when the true prevalence is unknown) and a confidence level of 95%, resulting in a required sample size of approximately 300 animals per species to estimate prevalence with a precision of ±5%−6%. Based on this calculation, a target sample size of approximately 300 dogs and 300 cats was established. Both symptomatic and asymptomatic animals were included in order to increase the probability of detecting positive cases and to better represent the investigated population. The point prevalence of positive samples among the total number of samples tested was calculated for each pathogen, with 95% confidence intervals estimated using the Wilson method. All analyses were performed using Stata, version 17.0 (StataCorp LLC, College Station, TX, USA).

## 3 Results

### 3.1 Sampled population

**Figure 1:**
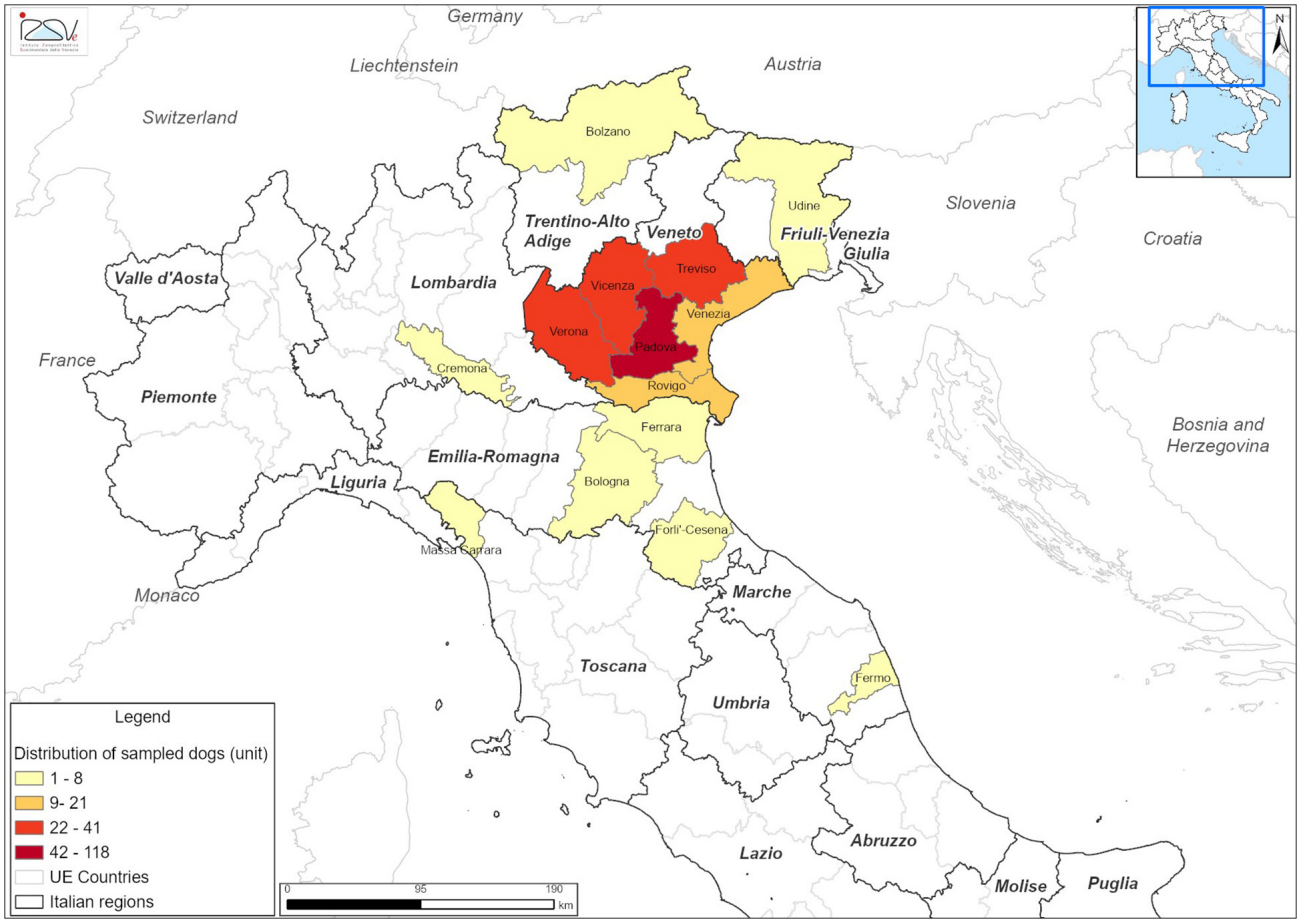
Geographical distribution of sampled dogs by province of origin in Italy between April 2023 and December 2025.

**Figure 2:**
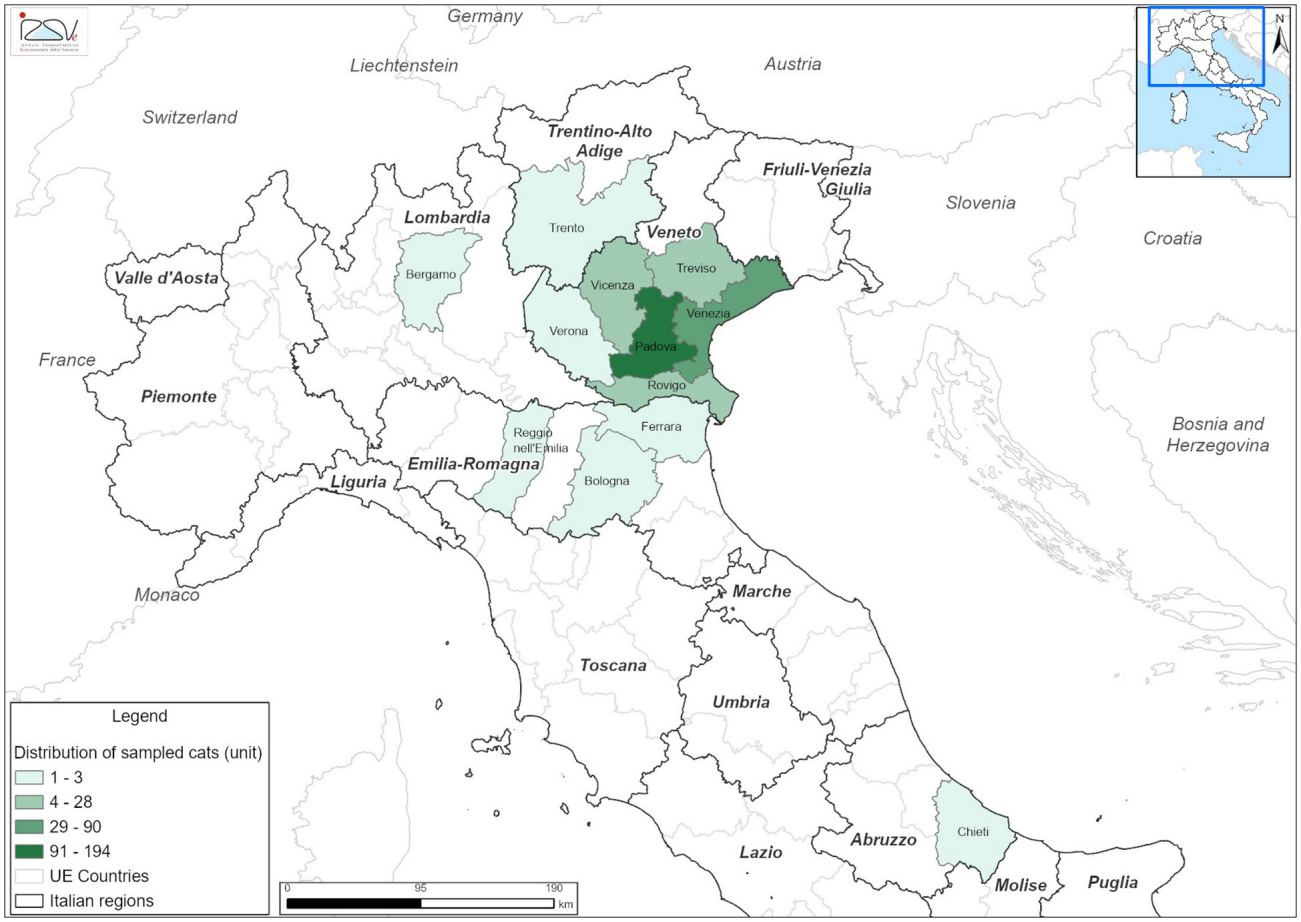
Geographical distribution of sampled cats by province of origin in Italy between April 2023 and December 2025.

A total of 648 fecal samples (278 from owned dogs and 370 from owned cats) were sampled between April 2023 and December 2025 as part of the RF MRV 21 research project. The animals originated primarily from nine provinces in North-East Italy: Bozen and Trento in the Trentino-Alto Adige region; Padua, Rovigo, Treviso, Venice, Vicenza, and Verona in the Veneto region; and Udine in the Friuli-Venice Giulia region. Additional samples were collected from areas outside the primary study region, including Bergamo and Cremona (Lombardia region), Bologna, Ferrara, Forlì-Cesena, and Reggio Emilia (Emilia-Romagna region), Fermo (Marche region), Massa-Carrara (Toscana region), and Chieti (Abruzzo region) (Figures 1, 2). The animals were categorized by age into four groups: < 1 years old (y/o), 1–4 y/o, 5–10 y/o, and >10 y/o. Among the dogs, females were marginally more prevalent (*n* = 142/278; 51.08%), whereas in the cat's population, males were slightly more represented (*n* = 201/370; 54.32%). At the time of sampling, most of both dogs (*n* = 151; 54.32%) and cats (*n* = 190; 51.35%) were asymptomatic. Detailed demographic and epidemiological data of the sampled population are summarized in Table 2.

**Table 2:** Demographic and epidemiological data of the sampled population.

	Dogs (278) N (%)	Cats (370) N (%)
Sex		
Male	136 (48, 92)	201 (54, 32)
M neutered	37 (13, 31)	104 (28, 11)
Female	142 (51, 08)	166 (44, 86)
F neutered	76 (27, 34)	83 (22, 43)
N.A	0 (0)	3 (0, 81)
Age		
< 1	27 (9, 71)	93 (25, 14)
1–4	92 (33, 09)	148 (40,00)
5–10	95 (34, 17)	62 (16, 76)
>10	52 (18, 71)	48 (12, 97)
N.A	12 (4, 32)	19 (5, 14)
Clinical symptoms		
Asymptomatic	151 (54, 32)	190 (51, 35)
Gastrointestinal	127 (45, 68)	180 (48, 65)
Provinces		
Bergamo	0 (0)	2 (0, 54)
Bologna	8 (2, 88)	2 (0, 54)
Bolzano	1 (0, 36)	0 (0)
Chieti	0 (0)	1 (0, 27)
Cremona	2 (0, 72)	0 (0)
Forlì-Cesena	2 (0, 72)	0 (0)
Ferrara	4 (1, 44)	2 (0, 54)
Fermo	3 (1, 08)	0 (0)
Massa-Carrara	1 (0, 36)	0 (0)
Padova	118 (42, 45)	194 (52, 43)
Reggio Emilia	0 (0)	1 (0, 27)
Rovigo	15 (5, 40)	20 (5, 41)
Trento	0 (0)	1 (0, 27)
Treviso	31 (11, 15)	23 (6, 22)
Udine	3 (1, 08)	0 (0)
Venezia	21 (7, 55)	90 (24, 32)
Vicenza	41 (14, 75)	26 (7, 57)
Verona	27 (9, 71)	3 (0, 81)
N.A	1 (0, 36)	3 (0, 81)

N.A, not available.

### 3.2 Molecular investigation results

Two out of 278 dogs (0.72%, 95% CI: 0.20–2.58) tested positive for MRV. Both were asymptomatic one-year-old males originating from the province of Padua. Among cats, three of the 370 samples (0.81%, 95% CI: 0.28–2.36) tested positive for MRV: two from females and one from a male. One four-year-old female was asymptomatic and originated from the province of Bologna, whereas the second female, aged 13 years, presented with diarrhea and originated from the province of Treviso. The male, younger than one year, was asymptomatic and originated from the province of Padua. RVA was detected in four dogs (1.44%, 95% CI: 0.56–3.64), all presenting with diarrhea and aged between 1 and 5 years old, three of them belonged to the same household. One dog originated from the province of Treviso and three from the province of Vicenza. RVA was also detected in seven cats (1.89%, 95% CI: 0.92–3.85) aged up to 1 year; six presented with diarrhea, whereas one was asymptomatic. Four cats originated from the province of Padua and three from the province of Venice. An overview of MRV and RVA positive animals, associated clinical signs, and detection of other viral and parasitic pathogens is provided in Table 3.

**Table 3:** Clinical and epidemiological data of MRV- and RVA-positive dogs and cats, detected by Real-time RT-PCR, including associated clinical and anamnestic findings, and co-detection of parvoviruses (CPV-2/FPV, Real-time PCR), coronaviruses (CcoV/FCoV, Real-time RT-PCR), and results of copromicroscopic examination.

ID	Species	Signalment	Area	Life habits	Clinical findings	Positivity				
						MRV	RVA	CPV-2 FPV	CCoV FCoV	Parasites
24DIA-PD/40118	Dog	Border collie M Age: 1 y/o, 3m/o	Urban- city	Indoor outdoor close contact with the owner	None	**POS**	NEG	NEG	NEG	NEG
24DIA-PD/40165	Dog (^*^)	English pointer M Age: 10 m/o	Rural	Outdoor (hunting dog)	Diarrhea (recurrent - mild severity) onset: chronic (>20 days)	NEG	**POS**	NEG	**POS**	**POS** **(*****Capillaria*****)**
24DIA-PD/40166	Dog (^*^)	English pointer M Age: 10 m/o	Rural	Outdoor (hunting dog)	Diarrhea (recurrent - mild severity) onset: chronic (>20 days)	NEG	**POS**	NEG	**POS**	NEG
24DIA-PD/40169	Dog (^*^)	English pointer M Age: 5 y/o	Rural	Outdoor (hunting dog)	Diarrhea (recurrent - mild severity) onset: chronic (>20 days)	NEG	**POS**	NEG	**POS**	NEG
25DIA-PD/40183	Dog	Bernese mountain F Age: 3 y/o	Rural	Indoor outdoor	Diarrhea, hematochezia onset: acute	NEG	**POS**	**POS**	NEG	NEG
25DIA-PD/40211	Dog	Mixed breed M Age: 1 y/o, 4m/o	Urban- city	Indoor outdoor close contact with the owner	None	**POS**	NEG	NEG	NEG	NEG
24DIA-PD/41169	Cat	DSH FS Age: 4 y/o	Rural	Outdoor indoor	None	**POS**	NEG	NEG	NEG	**POS** ***(Coccidia)***
24DIA-PD/41179	Cat	DSH FS Age: 3 y/o	Rural	Outdoor	Diarrhea onset: acute	**POS**	NEG	NEG	NEG	NEG
25DIA-PD/41202	Cat (□)	DSH M Age: 8 m/o	Rural	Outdoor indoor	Nausea, vomit, diarrhea onset: acute	NEG	**POS**	NEG	**POS**	NEG
25DIA-PD/41203	Cat (□)	DSH M Age: 8 m/o	Rural	Outdoor indoor	Nausea, vomit, diarrhea onset: acute	NEG	**POS**	NEG	**POS**	NEG
25DIA-PD/41204	Cat (□)	DSH M Age: 5 m/o	Rural	Indoor	Nausea, vomit, diarrhea onset: acute	NEG	**POS**	NEG	**POS**	NEG
25DIA-PD/41230	Cat (▴)	Siberian MN Age: 1 y/o	Urban- city	Indoor	Melena, lymphadenopathy onset: acute	NEG	**POS**	NEG	**POS**	NEG
25DIA-PD/41231	Cat (▴)	Siberian FS Age: 1 y/o	Urban- city	Indoor	Melena onset: acute	NEG	**POS**	NEG	**POS**	NEG
25DIA-PD/41303	Cat (♦)	DSH M Age: 3 m/o	Rural	Outdoor indoor	Diarrhea, ocular discharge onset: acute	NEG	**POS**	NEG	NEG	**POS (** ***Coccidia*** **)**
25DIA-PD/41304	Cat (♦)	DSH M Age: 3 m/o	Rural	Outdoor indoor	None	NEG	**POS**	NEG	NEG	NEG
25DIA-PD/41375	Cat	DSH M Age: 2 m/o	Urban- city	Indoor	None	**POS**	NEG	NEG	**POS**	**POS (** ***Nematoda*** **)**

^*^, □, ▴, ♦: indicate groups of cohabiting animals that shared the same indoor and outdoor environment, diet, and general living conditions.

M, male; MN, male neutered; FS, female spayed; F, female. DSH, Domestic shorthair; y/o, year-old, m/o, months-old. Bold values indicate all positive results obtained from the analyses performed.

As part of the diagnostic panel, CPV-2 and FPV were investigated in fecal samples, reporting a low prevalence in both dogs (4/278; 1.44%, 95% CI: 0.56–3.64) and cats (8/370; 2.16%, 95% CI: 1.10–4.21). Among CPV-2 positive dogs, two were asymptomatic at the time of sampling, while the remaining two showed severe gastrointestinal signs, including diarrhea and hematochezia; one of these dogs was also positive for RVA. The other CPV-2 positive dogs tested negative for all additional pathogens included in the analytical panel. In cats, FPV positivity was mainly associated with clinical disease, since gastrointestinal signs were present in all positive animals except two at the time of sampling. FPV was detected as a single infection in most cats, with the exception of three animals that were co-infected with FCoV. Notably, CPV-2 and FPV positive animals were predominantly puppies or young animals, except for one dog aged 17, confirming the well-known age-related susceptibility to parvoviral infections. The investigation of CCoV and FCoV revealed a markedly higher prevalence in cats (131/370; 35.41%, 95% CI: 30.71–40.41) compared to dogs (28/278; 10.07%, 95% CI: 7.06–14.18). All CCoV-positive dogs were young animals, and the majority (20/28; 71.43%, 95% CI: 52.95–84.77) presented gastrointestinal signs. Additionally, several of these dogs were co-infected with RVA (3/28; 10.71%, 95% CI: 3.72–27.20) or tested positive at coprological examination (6/28; 21.43%, 95% CI: 10.26–39.55). In contrast, FCoV-positive cats encompassed all age groups, and slightly more than half of them (68/131; 51.91%, 95% CI: 43.54–60.15) were symptomatic. Among FCoV-positive cats, co-infections with other pathogens were frequently observed, including MRV (1/131; 0.76%, 95% CI: 0.13–4.17), RVA (5/131; 3.82%, 95% CI: 1.66–8.62), FPV (3/131; 2.29%, 95% CI: 0.79–6.51), and intestinal parasites detected by coprological examination (10/131; 7.63%, 95% CI: 4.20–13.44). Overall, no consistent co-infection patterns were observed among MRV-positive animals, except for two cases: a 2-month-old kitten presenting mild clinical signs and co-infected with MRV, FCoV, and nematodes, and an asymptomatic adult cat that tested positive for MRV and coccidia. In contrast, RVA positivity was frequently associated with concurrent CCoV or FCoV infection. An overview of all investigated pathogens, the analyses performed, the biological materials sampled, and the 95% confidence intervals (CI) is provided in Table 4.

**Table 4:** Pathogens investigated during the RF MRV 21 research project, type of analysis performed, biological material sampled, and 95% confidence intervals (CI).

Pathogen	Analysis	Biological sample	Dogs (278) N				Cats (370) N			
			**Negative**	**Positive**	**Total**	**% (95% CI) prevalence**	**Negative**	**Positive**	**Total**	**% (95% CI) prevalence**
Mammalian orthoreovirus	Real-time RT-PCR	Feces	276	2	278	0, 72 (0, 20–2, 58)	367	3	370	0, 81 (0, 28–2, 36)
Rotavirus A	Real-time RT-PCR	Feces	274	4	278	1, 44 (0, 56–3, 64)	363	7	370	1, 89 (0, 92–3, 85)
Parvovirus	Real-time PCR	Feces	274	4	278	1, 44 (0, 56–3, 64)	362	8	370	2, 16 (1, 10–4, 21)
Coronavirus	Real-time RT-PCR	Feces	250	28	278	10, 07 (7, 06–14, 17)	239	131	370	35, 41 (30, 71–40, 41)
Nematodes	Flotation	Feces	270	8	278	2, 88 (1, 47–5, 57)	343	27	370	7, 30 (5, 06–10, 41)
Coccidia	Flotation	Feces	267	11	278	3, 96 (2, 22– 6, 98)	356	14	370	3, 78 (2, 27–6, 25)
*Toxoplasma gondii*	Flotation	Feces	278	0	278	0 (0–1, 36)	368	2	370	0, 54 (0, 15–1, 95)

### 3.3 Viral isolation

**Figure 3:**
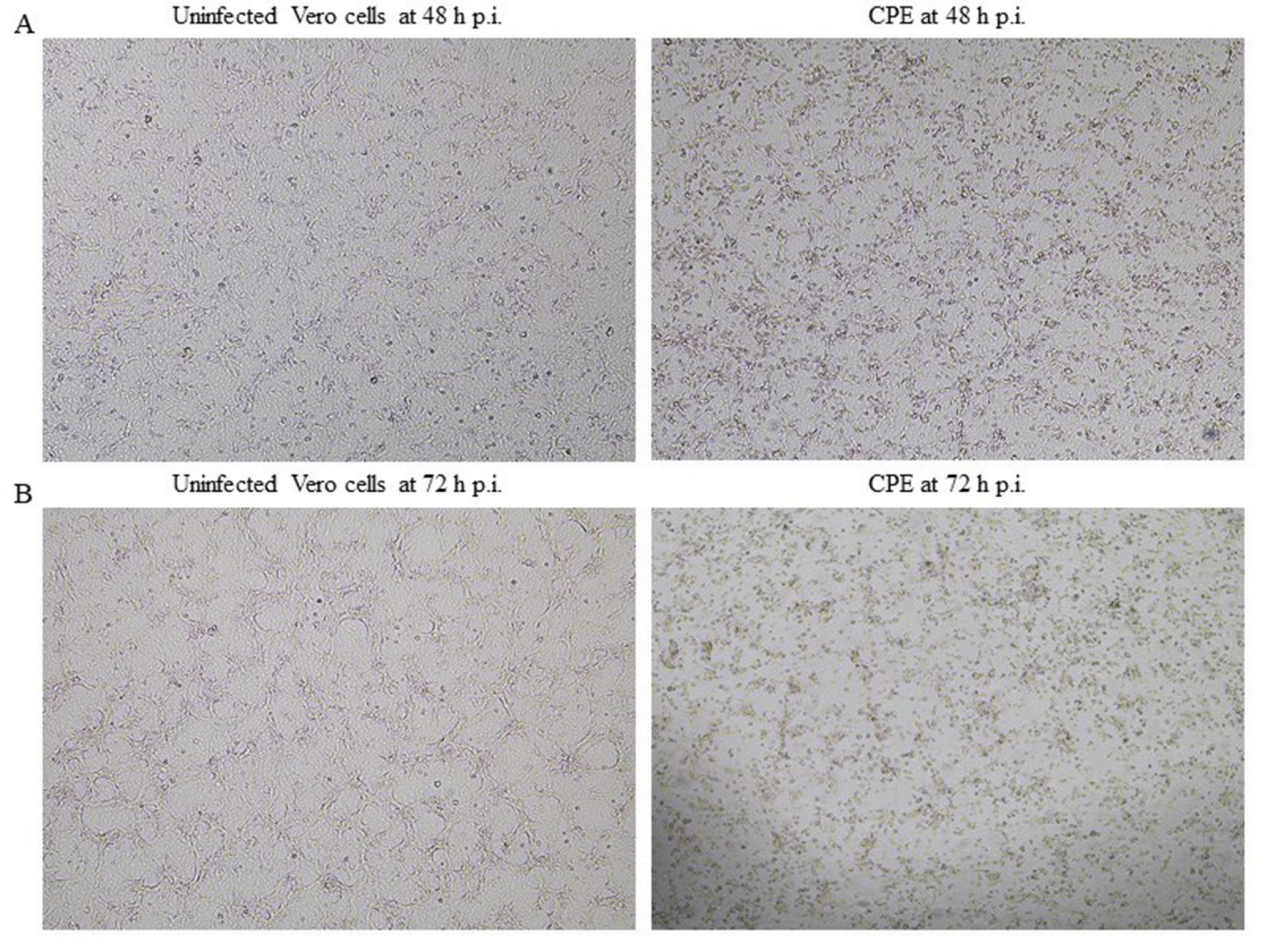
Uninfected Vero cells and cytopathic effect (CPE) observed in Vero cells infected with an MRV-positive fecal sample, visualized by inverted light microscopy at 40x magnification. **(A)** Uninfected Vero cells and CPE observed at 48 h post-infection (p.i.); **(B)** Uninfected Vero cells and CPE observed at 72 h p.i.

MRV isolation was attempted from all rRT-PCR positive samples (dogs: 2/278, 0.72%; cats: 3/370, 0.81%), as well as from MRV-positive samples identified in the previous study (dogs: 1/257, 0.39%; cats: 11/388, 2.83%). Successful MRV isolation in Vero cells was achieved for seven feline samples (four from RC IZSVE 12/19 and three from RF MRV 21) and one canine sample (RF MRV 21) (Table 5). MRV infection induced a characteristic CPE consisting of cell rounding, detachment, and subsequent lysis of the infected monolayer (Figure 3). In feline isolates, CPE became evident after the third passage, whereas the canine isolate exhibited clear cytopathic changes already after the first passage. When no visible CPE was observed, up to three blind passages were performed at weekly intervals. MRV was successfully isolated from three rectal swabs, one oropharyngeal swab, and two fecal samples collected from cats, while in dogs the virus was recovered exclusively from a fecal sample. The presence of MRV in all isolates was confirmed by molecular analysis and by whole-genome sequencing using next-generation sequencing (NGS). Detailed genomic characterization and phylogenetic analyses are beyond the scope of the present study and will be reported in a separate investigation. Virus isolation was also attempted for RVA-positive samples using different cell lines available in our laboratory (BGM, AGMK, CACO-2, MARC-145, and HRT-18), but it was unsuccessful.

**Table 5:** Samples testing positive for MRV by Real-time RT-PCR during the RC IZSVE 12/19 and RF MRV 21 projects and successfully isolated in Vero cells.

Project	Species	Sample ID
RC IZSVE 12/19	Cat	21DIA-PD/41049
	Cat	21DIA-PD/41051
	Cat	21DIA-PD/41117
	Cat	21DIA-PD/41162
RF MRV 21	Dog	25DIA-PD/40211
	Cat	24DIA-PD/41169
	Cat	24DIA-PD/41179
	Cat	25DIA-PD/41375

### 3.4 Copromicroscopic findings

Nineteen out of 278 dog fecal samples (6.83%, 95% CI: 4.42–10.43) tested positive for intestinal parasites. Detected parasites include nematodes (*n* = 8; 2.88%, 95% CI: 1.47–5.57) and coccidia (*n* = 11; 3.96%, 95% CI: (2.22–6.98). Twelve dogs presented gastrointestinal symptoms at the time of sampling; most were ≤ 2 years old, except for one aged four, two aged seven, and one aged nine years. Among cats, 43 out of 370 fecal samples (11.62%, 95% CI: 8.72–15.30) tested positive for intestinal parasites. One sample was unsuitable for analysis due to insufficient material. Positive samples included nematodes (*n* = 27; 7.30%, 95% CI: 5.06–10.41) and coccidia (*n* = 16; 4.32%, 95% CI: 2.64–7.15%), among which two cases of *Toxoplasma* sp. infection were identified. Nineteen cats showed gastrointestinal symptoms at the time of sampling; these animals were mostly ≤ 2 years old, except for one aged six years.

### 3.5 Statistics results

Descriptive analyses were performed to summarize epidemiological and clinical data associated with virus-positive animals. Prevalence estimates for each pathogen, calculated with 95% CI using the Wilson method, are summarized in Table 4. Due to the limited number of MRV and RVA-positive samples, no statistical analyses were performed to evaluate associations with demographic variables or co-infection with other pathogens, as such analyses would not provide reliable estimates.

## 4 Discussion

The present study provides update information on the detection of MRV and RVA in companion animals from different regions of Italy. Overall, MRV was detected in 0.77% of animals (5/648; 95% CI: 0.32%−1.78%), highlighting that while the virus can occur in domestic companion animals, its prevalence is very low. A lower prevalence was observed in owned animals (dogs: 2/278, 0.72%; cats: 3/370, 0.81%), compared with shelter animals, as previously reported in Italy (dogs: 1/257, 0.39%; cats: 11/388, 2.83%) (43). Compared with our previous results (43), in which MRV prevalence among shelter cats reached 2.83% (11/388), the lower prevalence observed in owned companion cats (0.81%; 3/370) may reflect differences in sampling approaches (oropharyngeal and rectal swabs vs. fecal samples) as well as differences in animal management conditions. Shelter environments are typically characterized by high population density and frequent animal turnover, conditions that may facilitate the transmission of pathogens. In the previous study, the higher prevalence was most likely influenced by the clustering of MRV-positive cats belonging to the same feline colony and sampled during a single session. In contrast, MRV-positive owned animals identified in the present study originated from different households and provinces. i Among all MRV positive animals, only a single 13-year-old female cat presented gastrointestinal symptoms, whereas all other infected animals (two dogs and two cats) were reported as asymptomatic at the time of sampling (Table 3). This observation is consistent with previous reports indicating that MRV infections are frequently associated with mild or asymptomatic clinical courses. However, given the limited number of positive cases, no conclusions regarding the clinical relevance of MRV infection can be drawn. Importantly, MRV isolates obtained from positive samples were confirmed by molecular assay and whole-genome sequencing.

RVA was detected in 11 out of 648 animals (1.70%; 95% CI: 0.95–3.02), including four dogs (1.44%) and seven cats (1.89%). Although the number of positive animals was limited, several RVA-positive animals presented gastrointestinal clinical signs, in contrast to MRV-positive animals that were mostly asymptomatic. These findings differ from our previous observations in shelter populations, where all RVA-positive animals were asymptomatic (dogs: 2/255, 0.78%; cats: 13/389, 3.34%) (43). This discrepancy may be partly explained by the sampling performed during the symptomatic phase, which could have amplified the reporting of clinical disease and increased the likelihood of RVA detection. However, due to the absence of a healthy control group and a comprehensive differential diagnosis panel, a causal association between RVA and the observed clinical signs cannot be established. These observations should therefore be considered hypothesis-generating and warrant further investigation in controlled studies. The detection rate of RVA in dogs observed in this study aligns with recent reports from Thailand (50, 51), although it was lower than the prevalence reported in other countries, including Germany (39.7%), Mexico (21%), and Brazil (8.2%) (46, 52, 53). RVA was detected in domestic cats at a low frequency (1.89%, 7/370), which is lower than that reported in other studies, such as those conducted in Germany (50.0%) and the United Kingdom (3.0%) (46, 54). The lower RVA prevalence observed in our study may be related to the fact that the sampled population consisted primarily of client-owned animals rather than shelter populations. Unlike shelters, which are characterized by high density, high turnover, and frequent introduction of new animals, household environments provide fewer opportunities for sustained transmission of pathogens. This structural difference plausibly accounts for the lower RVA detection rates in our cohort (55, 56).

In the present study, RVA-positive dogs were exclusively adult animals (5–10 years old), in contrast to previous reports in which RVA infection was predominantly detected in younger dogs (52). In cats, RVA detection occurred mainly in young individuals presenting gastrointestinal signs (8 months−3 years old). However, given the limited number of positive cases, these observations should be interpreted with caution.

Evidence from previous studies indicates that close cohabitation and shared environments may facilitate the transmission of viruses among animals housed in the same facility or colony (42, 57, 58). In the present study, some RVA-positive animals belonged to groups of cohabiting individuals, suggesting that environmental exposure and shared living conditions may contribute to localized transmission events. Nevertheless, further epidemiological investigations are needed to better clarify the transmission dynamics of these viruses in companion animal populations.

Monitoring these viruses in companion animals may help improve our understanding of their epidemiology and distribution in dogs and cats' populations. However, the low prevalence observed in the present study and the cross-sectional design do not allow conclusions regarding viral circulation dynamics or epidemiological significance.

Several limitations should be considered when interpreting the findings of this study. First, the number of MRV and RVA-positive animals was limited, which restricted the possibility of performing statistical analyses to evaluate potential associations with demographic variables or co-infection with other pathogens. In addition, another study limitation is represented by the choice of the diagnostic panel, that ideally could have included other collateral and more specific investigations, such as bacterial culture, assessment of bacterial virulence genes, and protozoal pathogens such as *Giardia* spp. We limited the panel, also for budget reasons, to the more common causes of gastrointestinal disorders, which are more often associated with clinical symptoms. This limitation would have been more significant in case of attempts to establish a statistical association with clinical symptoms and co-infections. Given the low prevalence of both MRV and RVA, statistical evaluations were extremely limited due to low significance.

Overall, the results of this study add new data on the detection of MRV and RVA in Italian dogs and cats and provide a basis for future investigations based on larger sample sizes and longitudinal study designs, which will be necessary to better clarify the epidemiology and potential clinical relevance of these viruses in companion animals. Further genomic characterization of the MRV isolates will also be necessary to better understand their genetic relationships. However, detailed genomic and phylogenetic analyses were beyond the scope of the present study and will be addressed in future investigations.

### 4.1 The role of co-infection

As part of the diagnostic panel, CPV-2 and FPV were investigated in fecal samples, revealing a low prevalence in both dogs (4/278, 1.44%) and cats (8/370, 2.16%). Among CPV-2 positive dogs, two were young animals (2 months to 4 years old) and asymptomatic at the time of sampling, whereas the remaining two, aged between 3 months to 1 year old, exhibited severe gastrointestinal signs, including diarrhea and haematochezia; one of these symptomatic dogs also tested positive for RVA. All other other CPV-2 positive dogs tested negative for all additional pathogens included in the analytical panel. In cats, FPV infection was detected predominantly in young individuals (< 1 year old), except for a single 17-year-old cat. FPV positivity was generally associated with clinical disease, as gastrointestinal signs were present in all but two cats at the time of sampling. FPV was detected as a single infection in most cats, except for three animals that were co-infected with FCoV. According with the literature, CPV-2 and FPV positive animals were predominantly puppies or young animals, confirming the age-related susceptibility to parvoviral infections (59–61). Parasitic infections were detected predominantly in younger subjects; however, no clear overlap was observed between MRV/RVA detection and parasitism in this dataset.

Current evidence suggests that MRV may act as a secondary or opportunistic pathogen within multifactorial disease processes rather than as a primary cause of illness. In line with this hypothesis, in the present study MRV was detected in two adult dogs, one adult cat, and a two-month-old kitten that were clinically healthy at the time of sampling, whereas clinical signs were observed in only one feline case, an elderly cat aged 13 years. Consistent with previous reports, RVA-positive animals in this study presented gastrointestinal clinical signs (33, 62). Overall, the low number of positive samples reported in our study, seem to be consistent with the well-established role of RVA as a primary enteropathogen, particularly in young animals, which constituted the majority of RVA-positive subjects in this study. In addition, no consistent co-infection patterns were observed among MRV-positive animals, whereas RVA positivity was frequently associated with concurrent CCoV/FCoV infection. Nevertheless, the high background prevalence of FCoV in cat populations, together with the limited number of RVA positive cases, does not allow any inference at this stage regarding potential epidemiological associations between these pathogens. Larger and more structured datasets will be required to clarify whether viral co infection may play a contributory role in the enteric disease of companion animals. As a study limitation, although additional tests could have expanded the range of detectable enteropathogens, the study design did not include an assessment of the role of coinfections or their contribution to clinical disease. The aim was limited to documenting the clinical and epidemiological context in which MRV and RVA were identified, rather than conducting a comprehensive differential diagnosis or exploring pathogen interactions. For this reason, and considering resource constraints, the panel focused on selected and common enteropathogens routinely investigated in diagnostic practice. This limitation would have been more relevant in a study designed to evaluate statistical associations between clinical signs and co infections, which was not the objective here and was not feasible given the low prevalence of both viruses.

## 5 Conclusions

This study demonstrates the presence of both Mammalian orthoreovirus (MRV) and Rotavirus A (RVA) in companion animals in Italy. The detection of these viruses in the investigated population fulfills the primary objective of the study, although they were identified at a low prevalence within the sampled population. Further investigations based on larger sample sizes, longitudinal study designs, and expanded genomic characterization will be necessary to better clarify the epidemiology and potential clinical relevance of these viruses in companion animal populations. Overall, this study provides additional data on the detection of MRV and RVA in Italian companion animals and offers a basis for future molecular and epidemiological investigations.

## Data Availability

The data supporting the findings of this study are available within the article. Requests for further details can be directed to the corresponding author.
